# A soil parameter dataset collected by agricultural farms in northern Greece

**DOI:** 10.1016/j.dib.2022.108408

**Published:** 2022-06-23

**Authors:** Panagiotis Tziachris, Vassilis Aschonitis, Eirini Metaxa, Areti Bountla

**Affiliations:** Soil and Water Resources Institute, Hellenic Agricultural Organization (H.A.O.)-DEMETER, Thessaloniki 57001, Greece

**Keywords:** Soil analyses, Soil properties, Northern Greece

## Abstract

In this dataset we present soil data analyses with properties such as pH, organic matter (OM), salinity (EC), etc., major elements (N, P, K, Mg) as well as some microelements (Fe, Zn, Mn, Cu, B) with significant impact on plant nutrition. The samples were collected from the top 30 cm of the soil depth through a period of 5 successive years. The survey area is in the regional unit of Grevena in northern Greece. This dataset can be used to evaluate the status of the soil for a variety of tasks.

## Specifications Table

**Table:** 

Subject	Soil science
Specific subject area	Soil chemical measurements
Type of data	Table
How data were acquired	Shallow (0–30 cm) soil samples were collected with a soil auger. Soils were dried on air. Extraction and determination of plant available nutrients were based on Soil Testing and Plant Analysis [1]
Data format	Raw
Parameters for data collection	Soil samples were collected during a soil study in period 2015-2019
Description of data collection	At each location, representative shallow soil samples were taken (2–3) close to each other and pooled together to make a composite sample. The minimum distances between two sampling points range around 50 m, and the average distance between points is 300 m.
Data quality	Quality of the provided data is checked through the WEPAL platform. Twice a year, four income soil samples are analyzed in determined parameters and the results are compared to those of other labs.
Data source location	Institution: Hellenic Agricultural Organization-Demeter/ Soil and Water Research Institute City: Thessaloniki Country: Greece Geographical borders of the survey area: from 40°01′50.76" N to 40°14′43.79" N from 21°17′30.06" E to 21°33′25.43" E
Data accessibility	Repository name: Soil data Grevena Data identification number: 10.17632/r7tjn68rmw.1 Direct URL to data: https://data.mendeley.com/datasets/r7tjn68rmw/1

## Value of the Data

• The presented data are mainly used by agricultural cooperatives and/or individual farmers, who want to know the soil properties to increase the yield, improve quality of products, reduce unnecessary inputs, and prevent a possible pollution for the surface and underground water.
• Raw soil data are useful to institutes or companies as a calibration dataset in their effort to design innovative tools, e.g., portable scanners for a fast, real time out soil monitoring in the field.
• Soil data could be used by fertilizers production industries for developing and improving their products.

## 1 Data Description

**Fig. 1:**
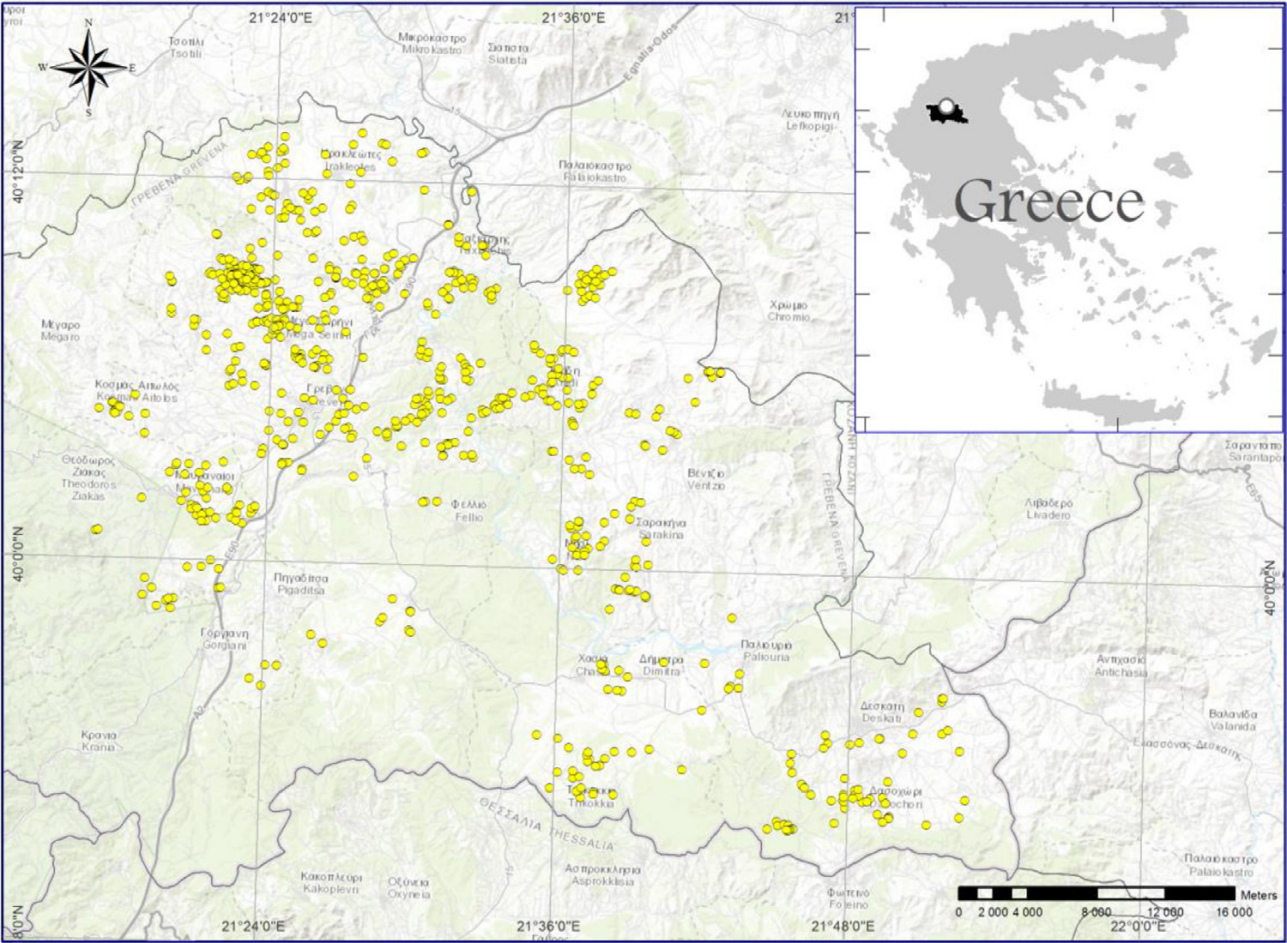
The 781 survey points and the study area (Grevena, Greece).

The data set contains raw soil data collected between 2015–2019. It consists of 781 survey points (Fig. 1). Each point consists of 16 soil parameters, total 12.480 data. This task was assigned to Soil and Water Research Institute (SWRI) by the Administrative Authorities of West Macedonia region, Greece. The altitude of the area ranges from approximately 500 m above sea level up to 900 m further north, and the area covers approximately 270 km^2^. The raw data set is contained in a XLSX file.

The raw data are used in an electronic database for constructing a digital soil map where the spatial soil variation is presented. The digital soil map is a useful tool for agronomists among other users who are increasingly seeking soil information that is more specific (soil properties) and more detailed (spatially clearer).

### 1.1 Example of Digital Soil Map Use Case

**Fig. 2:**
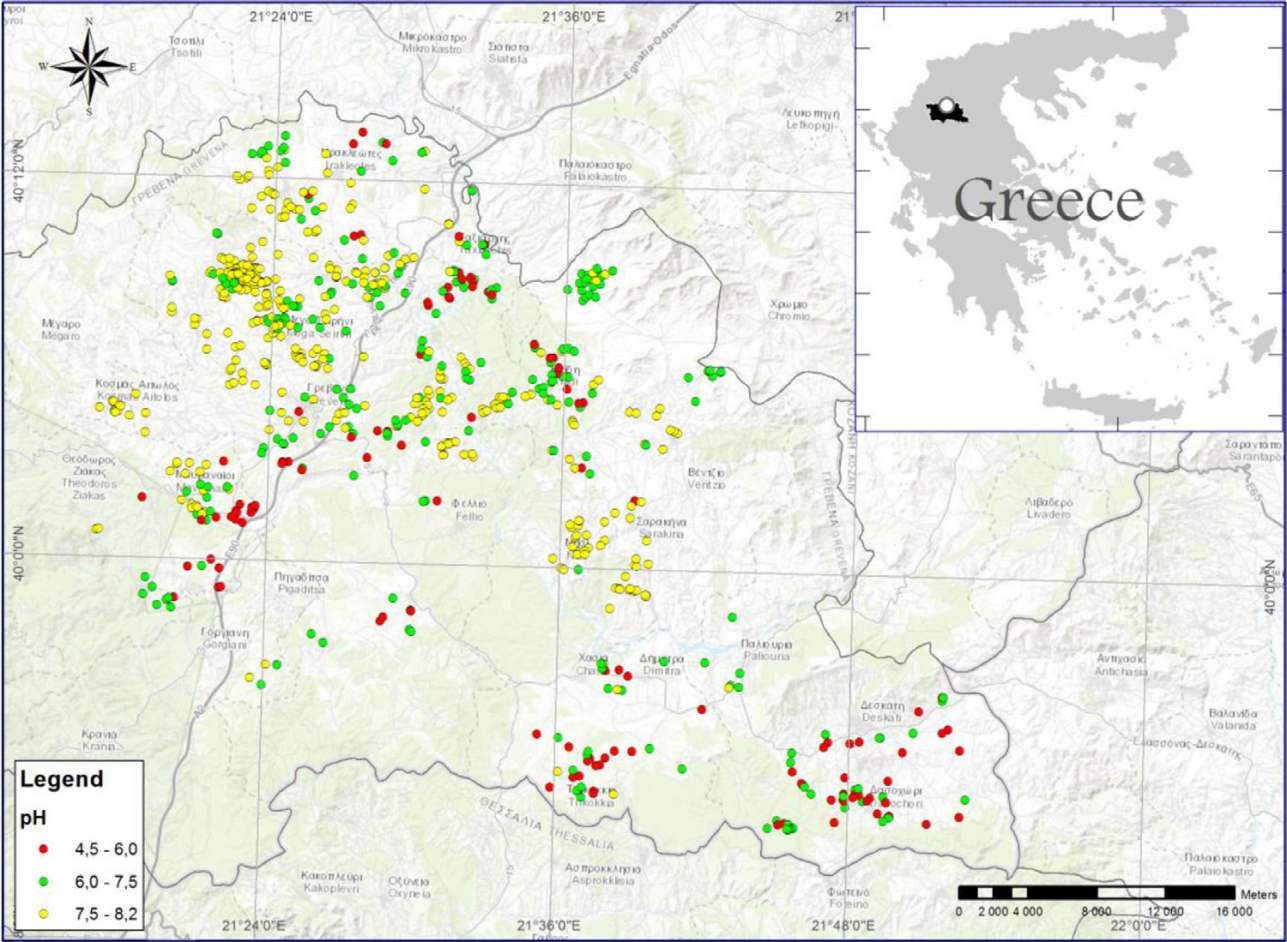
The pH digitized map classified in three levels (low pH 4.5-6.0/ median pH 6.0-7.5/ high pH 7.5-8.2).

A map of soil pH classification is presented in Fig. 2. It classifies the soil pH in three levels: (i) low, (ii) median, and (iii) high acidity. Low acidity corresponds to high pH values (>7.5), median acidity are pH values between 6.0–7.5 and high acidity corresponds to low pH values (4.5–6.0). This map can be used in multiple scenarios like for example to select the appropriate crop for establishment or to evaluate areas with pH problems of very high or low values and propose a suitable treatment. Soils with low or high pH can create challenges for growers, and impact yields and crop rotation options. For most crops, the ideal pH level is in the range of 6.0 to 7.0. Most nutrients are readily available at these levels, optimizing plant growth and improving crop competitiveness.

When soil pH is less than 6.0, the growth of acid sensitive crops, such as alfalfa, is reduced. Barley is moderately sensitive, and growth is affected when pH is less than 5.8. Trees, such as chestnut could be established only in acid soils up to 6.5 while most of the berries prefer high acidity until 5.2. On the contrary, truffle cultivation needs a low acidity with a pH value up to 8.

**Fig. 3:**
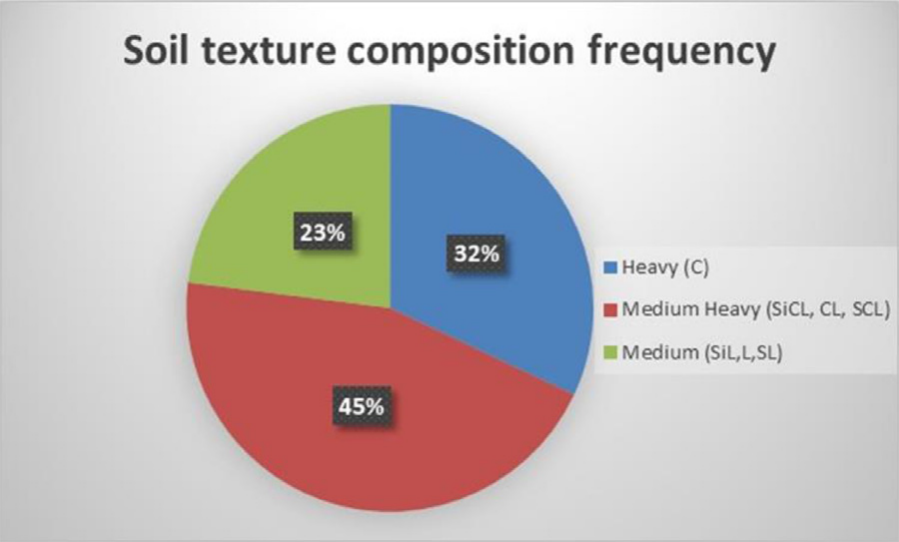
Frequency of soil texture composition.

**Fig. 4:**
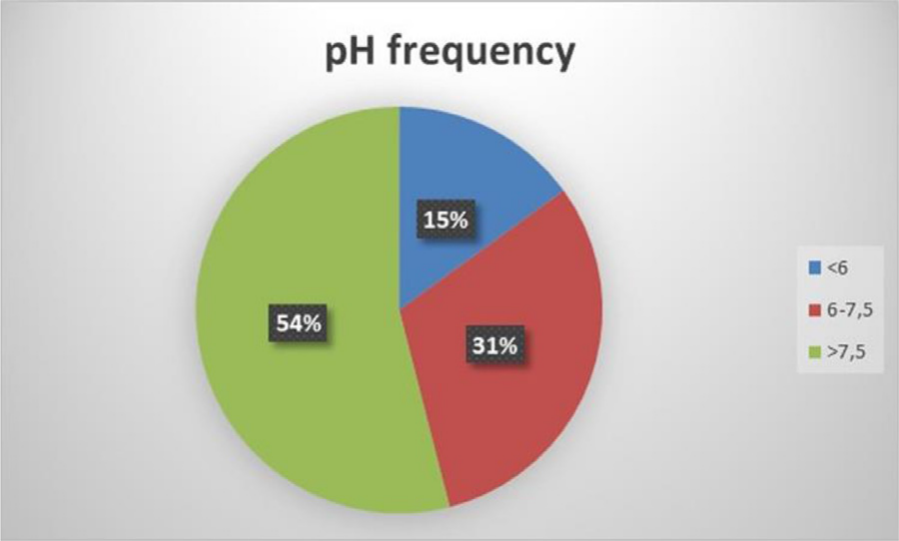
Frequency distribution of pH.

The data set in a processed form could offer valuable conclusions separately or with in interaction between the determined parameters. Table 1 presents some basic descriptive statistics of the measured soil data (See also Figs. 3 and 4)

**Table 1:** Descriptive statistics of selected parameters from the 781 locations in the Grevena area, Greece.

Variables	mean	SD	median	min	max
pH	7.13	0.90	7.55	4.49	8.20
O.M.	2.03	0.63	1.96	0.37	4.98
EC	0.45	0.30	0.40	0.13	5.62
CaCO_3_	13	13	9	0	78
N-NO_3_	12	11	9	0.2	121
P	14	15	9	1.1	152
K	296	194	255	31	1665
Mg	821	742	568	60	4836
Fe	31.20	21.01	19.87	4.63	187.94
Mn	15.87	15.17	10.10	2.38	189.21
Zn	0.67	0.68	0.48	0.10	7.09
Cu	2.03	2.46	1.48	0.21	33.91
B	0.38	0.22	0.35	0.10	1.90

### 1.2 Frequency Diagrams

Most of the soils in the area are heavy (C) and medium heavy (SiCL, CL, SCL) texture both reaching in a percentage up to 77%. A proportion of 23% is of medium texture (SiL, L, SL). Soils of light texture composition do not exist. In general, soils of medium texture are considered suitable for most of the crops. However, other applications such as the disposal of organic liquid waste produced of biogas plant could be applied in similar soils.

Most of the soils in the area (54%) are very alkaline (pH>7.5), thus are mostly suitable for acid sensitive crops. Acid soils are not so usual in the area (15%) and soils of medium acidity cover the rest 31% of the total dataset. In the latter category of medium acidity most of the crops can be productive, as concerned the pH value.

**Fig. 5:**
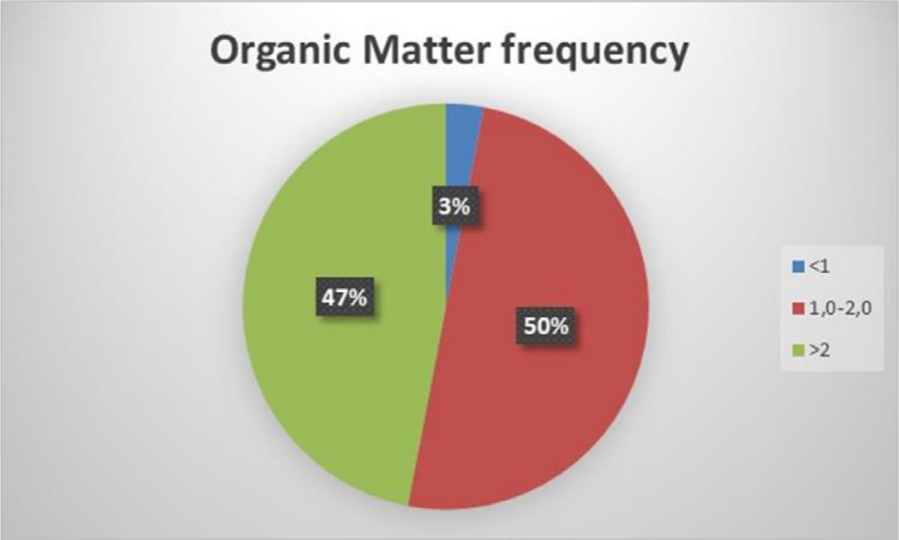
Frequency distribution of O.M.

For soil to be productive, organic matter should be higher than 2%. According to Fig. 5 about half of the soils (53%) have a low fertility level and need improvement.

**Fig. 6:**
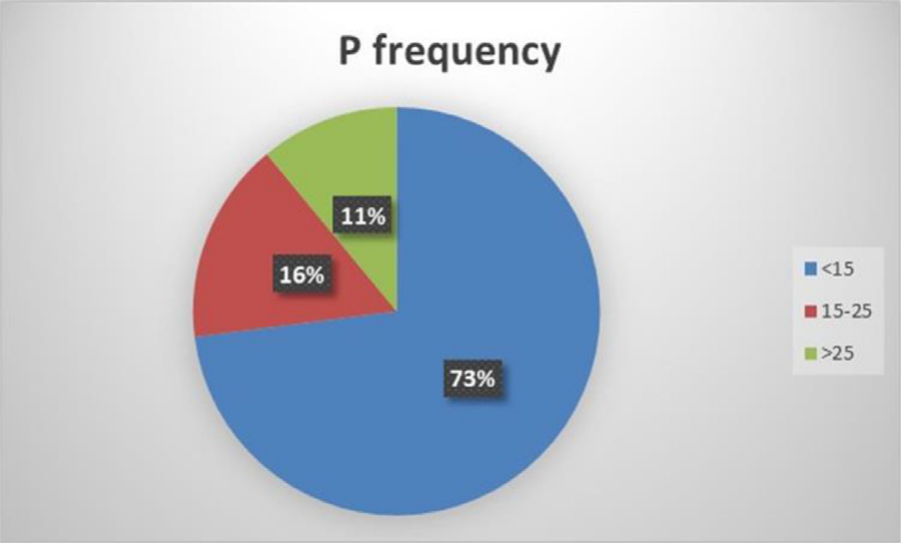
Frequency distribution of phosphorus.

Most of the soils in the area (73%) need addition of phosphate fertilizers to increase the P concentration more than the lower limit of 15 ppm, in the soil (Fig. 6).

Frequency distribution diagrams for each parameter provide a sufficient image of the soil variation in the area and could be used by agricultural cooperatives in scheduling on time the fertilization needs.

**Fig. 7:**
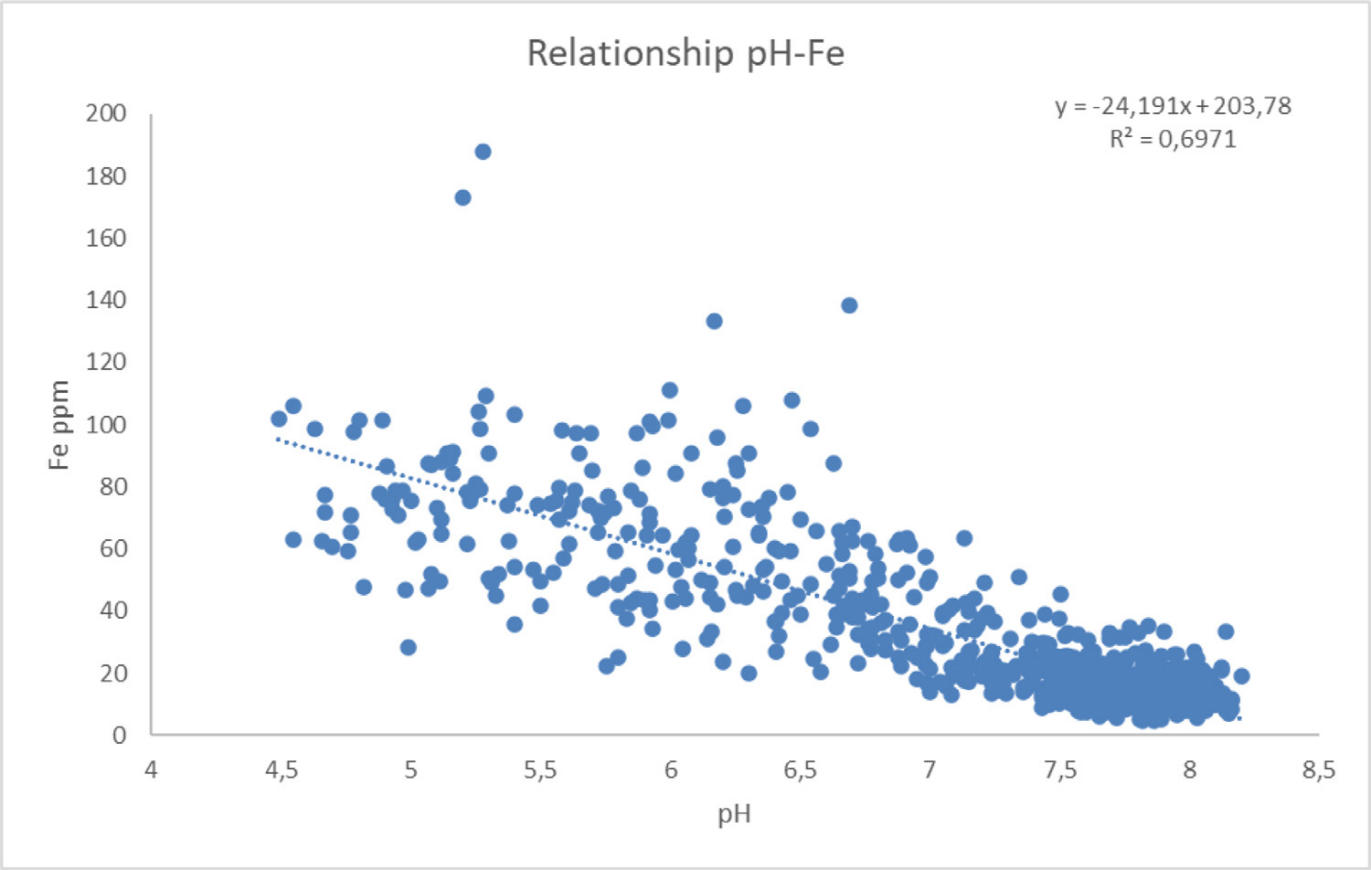
Linear regression diagram pH-Fe (Fe = -24.191*pH+203.78, R^2^=0.697).

**Fig. 8:**
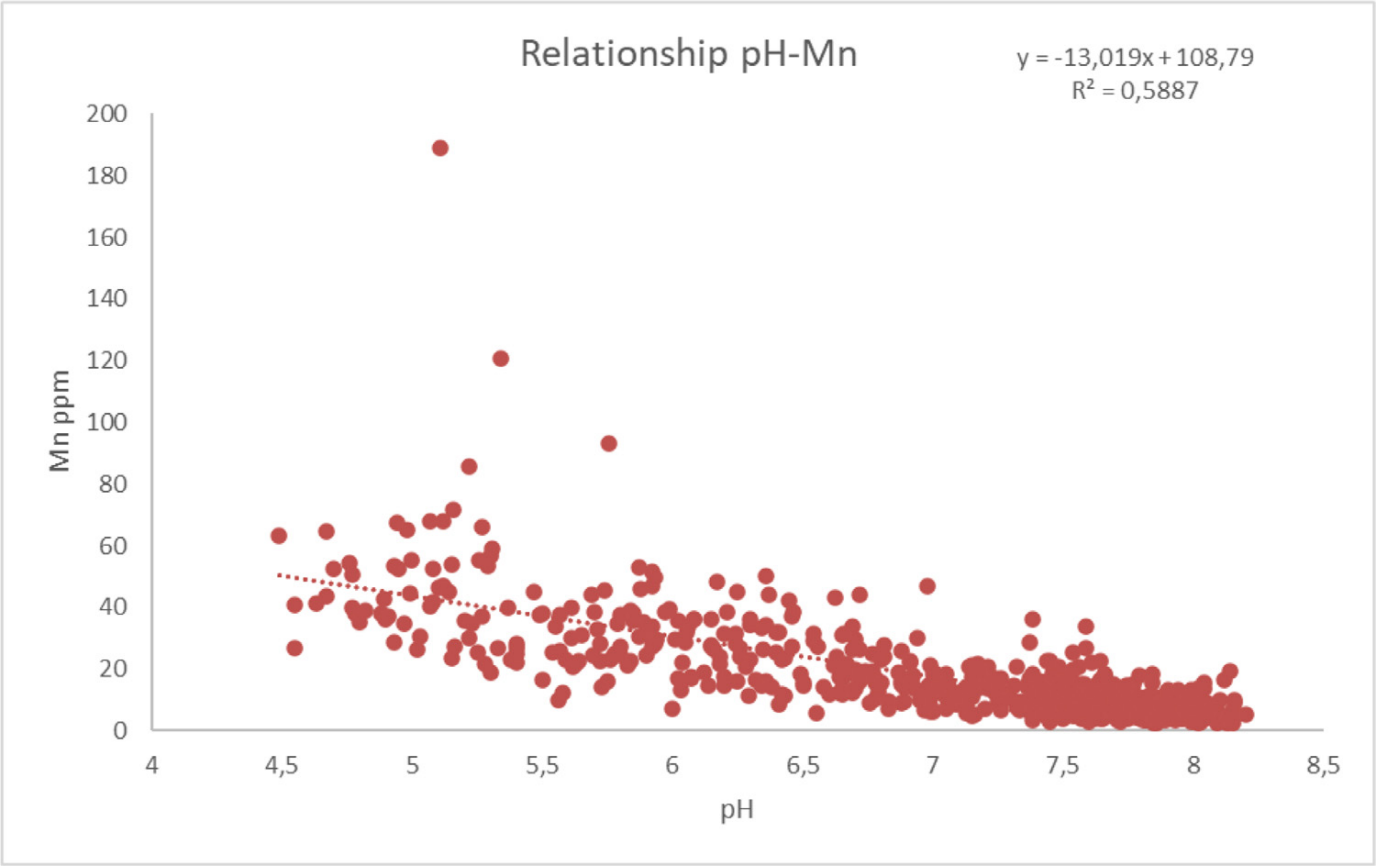
Linear regression diagram pH-Mn (Mn = -13.019*pH+108.79, R^2^=0.588).

Simple regression analysis between pH and metals of Fe (Fig. 7) and Mn (Fig. 8) has shown a statistically significant correlation in the study area. The existence of high correlation between soil properties and nutrients could influence the fertilization strategy in the area and be used for predicting soil parameters in a certain accuracy saving money and time.

## 2 Experimental Design, Materials and Methods

A soil survey was conducted for five years (2015–2019), mainly around autumn and early winter of each year. Specifically, 781 disturbed soil samples were obtained with a soil auger from depth 0–30 cm of the surface soil. At each location, representative samples were taken (2–3) close to each other and pooled together to make a composite sample. The minimum distances between two sampling points range around 50 m, and the average distance between points is 300 m.

The soil samples that were collected from the Grevena area were analyzed in the laboratory of the Soil and Water Resources Institute in accordance with Soil Testing and Plant Analysis and are presented in Table 2.

**Table 2:** Determined parameters.

	Parameters	Category	Unit	Method analyses	LOD⁎⁎
1	Clay (C)	Soil	%	Particle size analysis with hydrometer [2]	
2	Silt (Si)	Soil	%	Particle size analysis with hydrometer [2]	
3	Sand (S)	Soil	%	Particle size analysis with hydrometer [2]	
4	Electric conductivity (EC)	Soil	mS/cm	In soil saturation extract measured with conductometer [1]	
5	Acidity (pH)	Soil	—	In soil saturated paste measured with pH meter [3]	
6	Calcium Carbonate (CaCO_3_)	Soil	%	Acid neutralization method [4]	
7	Organic matter (O.M.)	Soil	ppm	Wet Oxidation measured with photometer at 600 nm [5]	
8	Nitrate Nitrogen (NO_3_-N)	Soil	ppm	With 2M KCl colorimetric with photometer [6]	0.5 ppm
9	Phosphorus (P)	Soil	ppm	With 0.5 M NaHCO_3_ pH 8.5 colorimetric with photometer [1]	0.01 ppm
10	Potassium (K)	Soil	ppm	With ammonium acetate at pH = 7.0 measured by ICP-OES [1]	
11	Magnesium (Mg)	Soil	ppm	With ammonium acetate at pH = 7.0 measured by ICP-OES [1]	
12	Iron (Fe)	Soil	ppm	DTPA* measured by ICP-OES [1]	0.0046 ppm
13	Zinc (Zn)	Soil	ppm	DTPA* measured by ICP-OES [1]	0.0059 ppm
14	Manganese (Mn)	Soil	ppm	DTPA* measured by ICP-OES [1]	0.0014 ppm
15	Copper (Cu)	Soil	ppm	DTPA* measured by ICP-OES [1]	0.0097 ppm
16	Boron (B)	Soil	ppm	Azomethine-H, colorimetric with photometer [7]	0.03 ppm

⁎ DTPA: Diethylenetriaminepentaacetic acid;

⁎⁎ LOD: Limits of Detection

## Ethics Statement

None.

## CRediT authorship contribution statement

**Panagiotis Tziachris:** Writing – review & editing. **Vassilis Aschonitis:** Data curation. **Eirini Metaxa:** Writing – original draft. **Areti Bountla:** Formal analysis.

## Declaration of Competing Interest

The authors declare that they have no known competing financial interests or personal relationships which have or could be perceived to have influenced the work reported in this article.

## Data Availability

Soil data Grevena (Original data) (Mendeley Data).
